# Monoclonal Antibodies: The Greatest Resource to Treat Multiple Myeloma

**DOI:** 10.3390/ijms24043136

**Published:** 2023-02-05

**Authors:** Fabiola De Luca, Alessandro Allegra, Carla Di Chio, Santo Previti, Maria Zappalà, Roberta Ettari

**Affiliations:** 1 Department of Chemical, Biological, Pharmaceutical and Environmental Chemistry, University of Messina, Viale F. Stagno d’Alcontres 31, 98166 Messina, Italy; 2 Department of Human Pathology in Adulthood and Childhood, University of Messina, Via Consolare Valeria, 90100 Messina, Italy

**Keywords:** multiple myeloma, cell apoptosis, monoclonal antibodies, immunotherapy

## Abstract

Multiple myeloma (MM) is a currently incurable hematologic cancer. This disease is characterized by immunological alterations of myeloid cells and lymphocytes. The first-line therapy involves the use of classic chemotherapy; however, many patients have a relapsed form that could evolve into a refractory MM. The new therapeutic frontiers involve the use of new monoclonal antibodies (Mab) such as daratumumab, isatuximab, and elotuzumab. In addition to monoclonal antibodies, new immunotherapies based on modern bispecific antibodies and chimeric antigen receptor (CAR) T cell therapy have been investigated. For this reason, immunotherapy represents the greatest hope for the treatment of MM. This review intends to focus the attention on the new approved antibody targets. The most important are: CD38 (daratumumab and isatuximab), SLAM7 (elotuzumab), and BCMA (belantamab mafodotin) for the treatment of MM currently used in clinical practice. Although the disease is still incurable, the future perspective is to find the best therapeutic combination among all available drugs.

## 1. Introduction

Multiple myeloma (MM) is a hematological cancer characterized by clonal expansion of plasma cells that accumulate in the bone marrow (BM) [1]. MM is the second most widespread blood cancer; it mostly affects people over the age of 60–65, with a slightly higher incidence in men than in women. Clinically it can present in a silent form or in an active form; the disease is often characterized by kidney failure or high levels of calcium in the blood because of the invasion of the bones by malignant cells. The increase in calcium levels interferes with the functions of the central nervous system and, consequently, can cause weakness and mental confusion. Despite the several currently available therapeutic alternatives, MM remains an incurable disease in which the patient becomes refractory to the previously used therapy after a variable period [2]. The immunological alterations that characterize the disease concern two immune populations: myeloid cells and lymphocytes.

It is highlighted for myeloid cells: an upregulation of inhibitory molecules by tumor cells, macrophages, and antigen presenting cells [3]; tumor-associated macrophages (TAMs) and myeloid-derived suppressor cells (MDSCs) [4,5], whereas dendritic cells (DCs) are functionally defective (due to the reduced expression of tumor antigens or HLA costimulatory molecules) [6]. The lymphocyte population causes upregulation of inhibitory ligands (such as PD-1 or TIGIT) [7,8]; infiltration of regulatory T and B lymphocytes through inhibition of T lymphocyte responses and promoting tumor growth [9,10]; mutated induction of lymphocyte responses (decreased lymphocytes or abnormal cytokines) [11,12]; and altered cell differentiation and antibody response of B lymphocytes [13].

In current clinical practice, although MM is currently incurable, several lines of treatment are available. In young patients (<65 years), autologous stem cell transplantation (ASCT) is the standard for new diagnoses [14]; pre-transplantation, high doses of melphalan are administered [15]. The average age of MM patients is about 70 years; the main problem for these patients is due to the fact that they are not eligible for transplantation (NTE), therefore, immunotherapy offers new lines of therapy with drugs that are effective and less toxic than those used in intensive treatment [16].

Currently, the standard of care for patients with NTE is melphalan–prednisone–bortezomib (VMP), lenalidomide and dexamethasone (Rd) and/or bortezomib, lenalidomide, and dexamethasone (VRd). However, new drugs have been tested both in the case of first-line treatment and relapsed disease. In fact, new generation immunomodulatory agents (IMiDs) (lenalidomide and pomalidomide) and proteasome inhibitors (PI) such as carfilzomib and ixazomib are available. In this review article, we will consider the currently used monoclonal antibodies (Mab) in clinical practice (daratumumab, isatuximab, and elotuzumab), together with several new drugs with novel mechanisms of action, such as modern bispecific antibody immunotherapies and CAR-T cell therapy, since immunotherapy represents the greatest hope for the treatment of MM [16].

**Figure 1:**
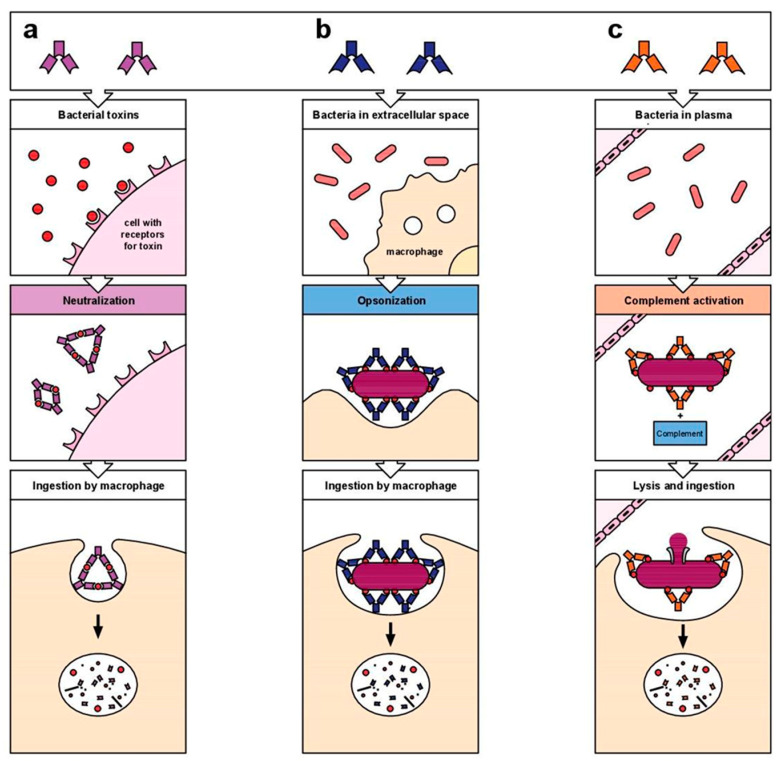
Mechanisms of action of antibodies: (**a**) antibody-dependent cell-mediated cytotoxicity (ADCC), (**b**) antibody-dependent cell phagocytosis (ADCP), and (**c**) complement-dependent cytotoxicity (CDC).

The use of monoclonal antibodies in clinical therapy has shown good results even for advanced patients [17]. In general, mAbs act with different mechanisms of action: antibody-dependent cell-mediated cytotoxicity (ADCC) leading to cancer cell death, antibody-dependent cell phagocytosis (ADCP), and through complement-dependent cytotoxicity (CDC) that activates a cascade of proteolytic enzymes (Figure 1) [18].

Recently, the FDA approved new antibodies targeting both CD38 (daratumumab and isatuximab) and SLAM7 (elotuzumab) for the treatment of MM [19].

Antibodies are proteins produced by the immune system in response to an antigenic stimulus. The invasion of an antigen stimulates the immune response: proliferation and differentiation of B lymphocytes in memory cells and plasma cells that are responsible for producing many soluble antibodies against the antigen. The specificity of the antibodies is high; they recognize foreign substances and fix and neutralize their effects. The portions of each antigen that interact specifically with their respective antibodies are called epitopes, each antibody recognizes a specific epitope of the immunizing antigen with high affinity. The structure of antibodies is characterized by two fundamental regions:

(1) Binding domain (varies from antibody to antibody): deputy to the antigen recognition;
(2) Effector domain (common to many different antibodies), which tends to destroy the antigen.

Structurally, they have two chains, a heavy (H) chain and two light (L) chains.

- Each chain contains a variable region (V) and a constant region (C); constant regions are held together by disulfide bonds.
- Both L and H chains consist of a variable NH_2_-terminal portion and a constant COOH-terminal.

In each of the variable regions there are three hypervariable sequences (CDR regions) that provide the molecular basis for the specificity of the antibody. The three complementarity regions, CDR1, CDR2, and CDR3, are located in correspondence to the variable regions (VH and VL) at the NH_2_-terminal ends of the two L chains and of the two H chains. Each antigen causes the production of many different antibodies; however, the great challenge has been the discovery of a cultivable cell line able to produce a single type of antibody molecule (monoclonal antibody) with a high affinity towards a specific epitope of an antigen that can be used in cancer treatment or in other types of incurable diseases.

**Figure 2:**
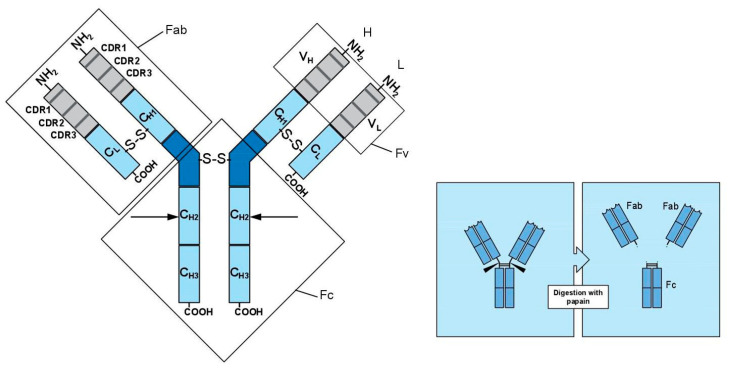
Structure of monoclonal antibody.

Antibodies without added elements are called “naked” and are composed of two binding regions of the fragment antigen (Fab) and a crystallizable fragment region (Fc) responsible for the interaction with the antigen (Figure 2) [2].

Monoclonal antibodies are produced by recombinant DNA technology and can be classified in several ways. It is possible to recognize four types of monoclonal antibodies:

(1) Murine monoclonal antibodies (-momab) are the first to be produced, their administration encounters difficulties and inconveniences. The main limitation is their immunogenicity. In fact, from the first administration an immune response can occur in 50–80% of patients (HAMA response, Human AntiMouse Antibody) and repeated administrations significantly increase the HAMA response, which causes the immediate destruction of the subsequent doses of administered antibodies. This limits the therapeutic efficacy.
(2) Chimeric monoclonal antibodies (-ximab) are characterized by a mouse portion and some segments of human origin and are obtained through genetic manipulation. The variable regions are coded by a murine antibody, and the constant regions are coded by a human antibody in the chimeric genes. The product of the constructed gene is a chimeric immunoglobulin that possesses specificity for the antigen typical of the murine monoclonal antibody (Fv mouse) with attenuated immunogenicity in humans and the effector functions of human antibodies (human Fc). Also, for chimeric antibodies the continuity of administration is limited by the HAMA response.
(3) Humanized monoclonal antibodies (-zumab) are obtained by genetic manipulation, the CDR regions constitute the only segments of murine origin. The CDRs (CDR1, CDR2, and CDR3) of murine origin replace the CDRs from the human antibody. The obtained immunoglobulin has the specificity of binding for the antigen of the murine monoclonal antibody but all the other properties of the human antibody molecule.
(4) Human monoclonal antibodies (-mumab) are entirely derived from human cells, thus have improved tolerability towards multiple administrations. On the other hand, they have very high production costs.

In this review, we will study the use in clinical therapy of a human monoclonal antibody (daratumumab), a chimeric one (isatuximab), and a humanized one (elotuzumab).

## 2. Daratumumab

Daratumumab, a naked monoclonal antibody, is the first monoclonal antibody directed against the CD38 antigen. The role of daratumumab in the treatment of MM is well established across multiple therapeutic lines, leading the way for the development of other inhibitors targeting CD38. It was approved by the FDA in November 2015 and by the EMA in May 2016 for the treatment of adult patients with multiple myeloma:

- In combination with lenalidomide and dexamethasone in patients newly diagnosed ineligible for autologous stem cell transplantation and in patients with relapsed or refractory MM who received at least one prior therapy;
- In combination with bortezomib, melphalan, and prednisone in newly diagnosed patients unsuitable for autologous stem cell transplantation;
- In combination with bortezomib, thalidomide, and dexamethasone in newly diagnosed patients eligible for autologous stem cell transplantation;
- In combination with bortezomib and dexamethasone in patients who received at least one previous therapy;
- In combination with pomalidomide and dexamethasone in patients who received at least two previous therapies including lenalidomide and a proteasome inhibitor;
- As monotherapy in patients who received at least three prior lines of therapy including a PI and an immunomodulatory agent or who are doubly refractory to a PI and an immunomodulatory agent [2,20].

The recommended dosage of daratumumab is 16 mg/kg body weight administered as an intravenous infusion. As for the dosing schedule, in combination with bortezomib and dexamethasone it is weekly in the first 9 weeks, and then every three weeks from week 10 to week 24. Finally, every four weeks until disease progression.

Recently, daratumumab was approved as first line for all MM patients and for amyloidosis. In fact, in the phase III ANDROMEDA trial in adults with newly diagnosed systemic AL amyloidosis, the addition of daratumumab to bortezomib, cyclophosphamide, and dexamethasone remarkably augmented the percentage of subjects attaining a hematological complete response relative to bortezomib, cyclophosphamide, and dexamethasone alone [21].

It is a human IgG1κ monoclonal antibody (mAb) that binds to the highly expressed CD38 transmembrane glycoprotein on the surface of MM cells and in other types of cells and tissues. The CD38 protein has multiple functions, such as receptor-mediated adhesion, signal transduction activity, and enzymatic activity. Daratumumab was shown to be a potent inhibitor of the in vivo and in vitro growth of CD38-expressing tumor cells to lead to the immune-mediated death of the tumor cells through multiple effector functions. Several studies suggest that daratumumab may induce tumor cell lysis in CD38-expressing tumors by CDC, ACDD, and ADCP. Subsets of myeloid-derived suppressor cells (CD38 + MDSCs), regulatory T cells (CD38 + Tregs), and B cells (CD38 + Bregs) are reduced by daratumumab-mediated cell lysis. T cells (CD3+, CD4+, and CD8+) are also known to express CD38 depending on their stage of development and level of activation. There is also a significant increase in absolute CD4+ and CD8 + T cell counts and percentage of lymphocytes in peripheral whole blood and bone marrow and an increase of T cell clonality, indicating immunomodulatory effects. Thus, together with immune-mediated cytotoxicity, anti-CD38 mAbs have an immunomodulatory effect that is dependent on the regulation of immune cells. Remarkably, augmented effector T cells also display improved killing ability due to increased concentrations of granzyme B, which stimulates caspases and activates programmed cell death. In animal experimental models, anti-CD38 mAbs administration caused greater concentrations of NAD+ in T effector cells, augmenting their antitumor effects. These findings help to clarify the clinical results for daratumumab, and its use appears to be a possible strategy for affecting practically all tumor subclones [22].

Daratumumab induces apoptosis in vitro and modulates the enzymatic activity of CD38, inhibiting the cyclase activity of the enzyme and stimulating the hydrolastic activity [1,22,23,24,25]. Several studies demonstrate the cytotoxic activity of daratumumab both in vitro and in vivo. Indeed, CD38 levels in pretreated MM patients could act as a biomarker of response against the anti-CD38 mAb daratumumab [1,23]. Daratumumab is administered intravenously (Dara iv), the infusion lasts about 7 h for the induction infusion and 4 h for the following ones [2,20]; however, a subcutaneous formulation (Dara sc) is also available starting from December 2021 [26,27].

When compared with the subcutaneous formulation, intravenous daratumumab treatment necessitates longer infusion periods and is linked to a greater incidence of infusion-related reactions (IRRs). Several nations, including the USA and the EU, have approved the use of SC daratumumab co-formulated with recombinant human hyaluronidase in combination with bortezomib, cyclophosphamide, and dexamethasone for the treatment of adult patients with multiple myeloma [28].

The COLUMBA study, an open-label noninferiority clinical trial that randomized 263 patients to daratumumab and hyaluronidase-fihj and 259 patients to intravenous daratumumab, served as the foundation for the FDA’s approval of the new formulation of daratumumab and hyaluronidase-fihj (monotherapy). The overall response rate (ORR) and the maximal C^trough^ on cycle 3 day 1 predose were the co-primary end goals in this study. For the two end goals, daratumumab and hyaluronidase-fihj were comparable to daratumumab IV [29].

Daratumumab demonstrated an excellent safety profile in both formulations for both patients with relapsed/refractory multiple myeloma and patients with newly diagnosed multiple myeloma (eligible/ineligible for transplant), together with a high rate of infusion reactions. However, it has been demonstrated that daratumumab binds to CD38 on erythrocytes and provokes pan agglutination in indirect antiglobulin tests (IATs), causing false positives for the IAT [30,31]. Most myeloma patients will require RBC transfusions due to the high prevalence of bone marrow suppression and anemia following chemotherapy or during bone marrow transplantation. For this reason, patients should perform RBC antigen phenotypic screening before transfusion; additionally, the blood bank should be informed when scheduling blood sessions that patients have received anti-CD38 monoclonal antibodies. Dithiothreitol (DTT), proteolytic enzymes, and CD38-deficient RBCs from neonatal cord blood may be used, if possible, to process reagent RBC in the IAT; papain, excessive soluble CD38 protein, and anti-CD38 monoclonal antibodies may be used to interfere with anti-CD38 monoclonal antibodies in serum; and RBC antigen genotyping may be carried out before transfusion. DTT is currently the approach that is used the most [32].

Furthermore, the standard of care for patients with newly diagnosed multiple myeloma who are transplant eligible is high-dose therapy followed by autologous stem cell transplantation. After an autologous transplant, a sufficient stem cell yield is necessary for prompt hematopoietic reconstitution. In transplant-eligible patients with newly diagnosed MM, the phase III CASSIOPEIA study compared daratumumab with the standard-of-care regimen bortezomib/thalidomide/dexamethasone (D-VTd) to bortezomib/thalidomide/dexamethasone (VTd). Although the D-VTd group used more plerixafor and had a lower stem cell yield in this trial, the addition of daratumumab to VTd did not affect the feasibility, safety, or success of engraftment after transplant. Possible motives why daratumumab provokes a lower stem cell yield are unknown; however, daratumumab may produce some forms of interference via an undetermined mechanism, as CD34+-committed stem cells present a low concentration of CD38 [33].

In vitro studies displayed a clear synergism between anti-CD38 mAbs (daratumumab and isatuximab) and IMiDs (lenalidomide and pomalidomide), principally owing to an augmented NK activity caused by IMiDs that enhances both the number and activity of NK cells and subsequently ADCC, as well as the cytotoxic effect of macrophages, thus augmenting ADCP [34]. This finding stimulated the investigation of the in vivo effect of the addition of anti-CD38 mAbs to IMiD-based combinations. Anti-CD38 mAbs also demonstrated an additive effect with PIs [35], although the precise mechanisms are less clear. Finally, several mechanisms of resistance to anti-CD38 MoAbs have been reported, comprising direct cellular apoptosis, antibody-dependent cellular cytotoxicity, antibody-dependent cellular phagocytosis, or complement-dependent cytotoxicity failure. High expression quantities of the CD38 antigen on MM PCs are indispensable for anti-CD38 activity [36]; however, during daratumumab administration, CD38 expression reduces via JAK-STAT3 signaling pathway alterations [37] and return to basal concentrations 3–6 months after daratumumab suspension. Thus, neoplastic clones presenting low CD38 expression could proliferate during therapy and cells such as granulocytes and monocytes might promote the immune escape of neoplastic PCs [38]. CD38 reduction could also be supported by the inhibitory effects of microRNAs (miRNA), such as miRNA-26a [39]. Use of anti-CD38 factors also regulates the expression of genes implicated in metabolism management and cell cycle phenomena [40]. Finally, the occurrence of drug antibodies may nullify the effects of CD38 antibodies. However, neutralizing anti-daratumumab and anti-isatuximab antibodies have not been found in treated MM subjects to date [41].

## 3. Elotuzumab

Elotuzumab is an immune-stimulating humanized IgG1 naked mAb used in clinical practice that was approved by the FDA in November 2015 and by the EMA in January 2016:

- In combination with lenalidomide and dexamethasone for the treatment of MM in adult patients who received at least one prior line of therapy;
- In combination with pomalidomide and dexamethasone for the treatment of adult patients with relapsed and refractory MM who received at least two lines of therapy (including lenalidomide and a proteasome inhibitor) and with disease progression [42].

The molecular target of elotuzumab is the surface glycoprotein SLAMF7 (signaling the lymphocytic activating molecule family 7), which is mainly expressed by NK and normal or tumor plasma cells where it promotes growth and survival [43]. The mechanism of action of elotuzumab occurs by blocking the interactions that lead to the growth and survival of cancer cells. Moreover, it stimulates NK cells by enhancing their ADCC activity [44,45].

Elotuzumab directly activates NK cells through SLAMF7 and Fc receptors, enhancing their anti-myeloma activity in vitro. SLAMF7 is present on myeloma cells, and it promotes cell death through the interaction with the Fc receptors placed on specific cells of the immune system.

NK cell-mediated antibody dependent (ADCC) and macrophage-mediated antibody-dependent cell phagocytosis (ADCP) [42,45]. The use of elotuzumab alone is limited by the fact that patients with RRMM demonstrate impairment of NK cells with a decrease in efficacy. In contrast, the combination with lenalidomide or pomalidomide and dexamethasone demonstrated better clinical efficacy [1,46,47]. The recommended dose of elotuzumab is 10 mg/kg body weight administered intravenously every week on days 1, 8, 15, and 22 for the first two treatment cycles and every 2 weeks thereafter on days 1 and 15. The length of each treatment cycle is 28 days [42]. Treatment should continue until disease progression or unacceptable toxicity. When administered with lenalidomide, the ORR was 78.5%, with a median OS in months of 48.3. An interesting aspect of this monoclonal antibody concerns its safety, as it has shown a low rate of reactions at the time of infusion and no further toxicity; therefore, it is also indicated for frail patients [2,42]. However, it has not shown encouraging results for patients with newly diagnosed multiple myeloma [2,48]. As for the onset, the success of immunotherapy depends on the normal function of immune cells, mainly T cell function, comprising identification, chemotaxis, and the control of killing by cytokines. Recently, a study detected a reduction in the expression of CCL20 (chemokine (C-C motif) ligand 20 and macrophage infectious protein-3α, mip-3α) in the drug-resistant cells [49].

## 4. Isatuximab

Isatuximab is a naked chimeric mAb that has the same target as daratumumab, CD38, and similarly exhibits proapoptotic activity through the caspase-dependent apoptotic pathway and the lysosomal cell death pathway [1,2,50]. Isatixumab was approved by the FDA in March 2020 and by the EMA in June 2020:

- In combination with pomalidomide and dexamethasone for the treatment of adult patients with relapsed and refractory MM who received at least two previous therapies, including lenalidomide and a proteasome inhibitor, and with disease progression during the last therapy;
- In combination with carfilzomib and dexamethasone for the treatment of adult patients with MM who have received at least one previous therapy.

Isatuximab is an IgG1-derived monoclonal antibody that binds to a specific extracellular epitope of the CD38 receptor. In vitro, isatuximab acts through IgG Fc-dependent mechanisms, including ADCC, ADCP, and CDC and causes apoptosis with an Fc-independent mechanism. Furthermore, it can activate NK cells in the absence of CD38-positive target tumor cells. In patients treated with isatuximab alone, a decrease in absolute lymphocyte counts was observed in vivo, particularly for CD16+ and CD56 + NK cells, CD19 + B lymphocytes, CD4+ and TREG T lymphocytes (CD3+, CD4+, CD25+, CD127-) in peripheral blood. This indicates an adaptive immune response. Isatuximab is administered intravenously at a dose of 10 mg/kg [43].

The approval of isatuximab came after several phase 1 studies that evaluated its safety and efficacy both in combination [2,51] and as a monotherapy [52]. The phase 1b study TCD11863- NCT01749969, the primary objective of which was to determine the maximum tolerated dose, is an open-label, dose escalation study that evaluated the safety, efficacy, and pharmacokinetics of anti-CD38 monoclonal antibody administered in 2 schedules in patients with RRMM (Table 1). Another study, TCD14079–NCT02283775, evaluated isatuximab as a monotherapy for patients with RRMM (Table 1). The primary objective was to determine the maximum tolerated dose (MTD) of isatuximab. Two phase 2 studies, NCT01084252 and NCT02514668, evaluated the safety, pharmacokinetics, and efficacy of isatuximab in patients with RRMM based on the duration of response, clinical benefit rate, and progression-free survival [1]. However, the phase 3 studies that led to the approval of isatuximab were: ICARIA-MM/NCT02990338 and IKEMA-NCT03275285 (Table 1) [2,53,54]. The ICARIA-MM study is a randomized, multi-center, open-label, phase 3 study involving 102 hospitals. Eligible participants were adult patients with RRMM who had received at least two prior lines of treatment (including lenalidomide and a proteasome inhibitor) and were not previously treated with an anti-CD38 monoclonal antibody (Table 1). Treatment continued until disease progression, unacceptable toxicity, or withdrawal of consent. Dose reductions for adverse reactions were allowed for pomalidomide and dexamethasone but not for isatuximab. The primary endpoint was progression-free survival. Safety was assessed in all participants who received at least one dose of the study drug. The most frequent adverse events emerging from treatment (any grade: isatuximab–pomalidomide–dexamethasone vs. pomalidomide–dexamethasone) were infusion reactions, upper respiratory tract infections, and diarrhea. The addition of isatuximab to pomalidomide–dexamethasone significantly improves progression-free survival in patients with RRMM. Isatuximab is an important new treatment option for patients refractory to lenalidomide and a proteasome inhibitor [2,53]. IKEMA was a prospective, randomized, open-label, parallel group, phase 3 study conducted in 69 study centers. Adult RRMM patients who had received one to three prior lines of therapy and had measurable M-protein in serum or urine were eligible. Patients were randomly assigned to isatuximab plus carfilzomib–dexamethasone (isatuximab group) or carfilzomib–dexamethasone (control group). Treatment continued until progression or unacceptable toxicity. The primary endpoint was progression-free survival and was assessed in the intention-to-treat population based on assigned treatment. Safety was evaluated in all patients who received at least one dose based on the treatment received. In this study, 179 were randomly assigned to the isatuximab group and 123 to the control group. Adding isatuximab to carfilzomib–dexamethasone has been shown to significantly improve progression-free survival and depth of response in RRMM patients [54]. The pharmacokinetics of isatuximab were evaluated in 476 MM patients who received intravenous isatuximab as a monotherapy or in combination with pomalidomide and dexamethasone, 1 to 20 mg/kg, once weekly or every 2 weeks. Isatuximab has a nonlinear pharmacokinetic drug arrangement and is target-mediated in binding to the CD38 receptor [43].

## 5. ADC

In the field of immunotherapy, a new model has been developed and tested and also used for MM based on drug-conjugated antibodies (ADC) [1]. ADCs are a new class of agents, consisting of three elements: a tumor-specific monoclonal antibody (mAb), a cytotoxic molecule called a payload, and a specialized chemical linker that connects them. The antibody recognizes and binds the antigen on the tumor cell, the complex is internalized, and the conjugated drug is released, producing cytotoxicity and cell death. This type of new conjugated antibody aims to be directed only at tumor cells, sparing healthy ones, maximizing efficacy, and reducing systemic toxicity associated with chemotherapy alone [57,58]. The payloads used in ADCs are small molecules with high hydrophobicity [59,60]. An important parameter is the drug–antibody ratio (DAR), which is the average number of payload molecules attached to a single mAb. The ideal level of this parameter varies from drug to drug and affects the stability of the drug in the circulation, the ability to penetrate the tumor, the antitumor efficacy, and toxicity [61]. Payloads commonly used in ADCs can be divided into two main categories: microtubule inhibitors and DNA damaging agents. Two microtubule inhibitors currently used are maytansinoids and auristatins. Two auristatin derivatives are commonly used for ADC constructions: monomethyl auristatin E (MMAE, vedotin) and monomethyl auristatin F (MMAF, mafodotin) [58,62,63,64]. The ideal characteristics of a linker should not allow for premature deconjugation in the circulation, because this could cause a systemic toxicity. The linkers currently used are divided into cleavable and non-cleavable linkers. The former are sensitive to various intracellular conditions, whereas the latter are more stable but rely on complete proteolytic degradation by lysosomes to release active payloads [65].

## 6. ADCs Approved for Multiple Myeloma

Some conjugated antibodies with different structures, payloads, and antigen targets are under study. This is related to their versatility and efficacy for the treatment of relapsed or refractory MM [2,66].

A conjugated antibody currently used in clinical practice is belantamab mafodotin, which is directed to CD269, also known as B cell maturation antigen (BCMA). BCMA is a transmembrane receptor required for B cell maturation that is expressed on malignant plasma cells, functions as a tumor marker in MM, and is generally associated with a worse prognosis [67,68]. Belantamab mafodotin is a drug–antibody conjugate that contains belantamab, a humanized monoclonal IgG1k antibody produced by recombinant DNA technology in a mammalian (Chinese hamster ovary) cell line, that is conjugated to maleimidocaproyl monomethyl auristatin F (mcMMAF), which is a cytotoxic agent [69]. Belantamab mafodotin was approved by the FDA and EMA in August 2020 and is indicated as a monotherapy for the treatment of multiple myeloma in adult patients who have received at least four previous therapies and whose disease is refractory to at least one proteasome inhibitor, an immunomodulatory agent, and an anti-CD38 monoclonal antibody and showed disease progression during the last therapy. This conjugated antibody binds to BCMA on the cell surface and is rapidly internalized. Inside the tumor cell, the cytotoxic agent is released and causes cell cycle arrest and cell death. The antibody intensifies the recruitment and activation of effector immune cells, killing tumor cells through cellular cytotoxicity and antibody-dependent phagocytosis. Belanramab mafodotin is administered intravenously at a dose of 2.5 mg/kg every 3 weeks. In a study, the overall response rate (ORR) was 32% with a clinical benefit rate of 36% and a median duration of response of 11months.

The most reported adverse events were thrombocytopenia and keratopathy, possibly due to the lack of drug-specific absorption, independently of BCMA, in the corneal basal epithelial layer [69,70,71]. Based on encouraging results from preclinical studies in which the ability to improve the recruitment and activation of immune effector cells and increase the killing of tumor cells by antibody-dependent cellular cytotoxicity was highlighted [72], significant in vitro and in vivo activity against MM cell lines was confirmed [2], which led to clinical phase studies. The clinical phase studies led to the approval of belantamab mafodotin. The first of these was a phase I study called DREAMM-1 [73] NCT02064387, which had the aim of evaluating the safety and tolerability of monotherapy with GSK2857916 in patients with RRMM (Table 1). It was an international, multicenter, open label study. The study was divided into two parts, a dose escalation phase in Part 1 assessed safety, tolerability, and pharmacokinetics to identify the recommended Phase 2 dose. Part 2 assessed the safety, tolerability, pharmacokinetics, and preliminary clinical activity of the recommended dose of Phase 2 Part 1. The first study was followed by the DREAMM-2 [55] study in which safety and activity were further investigated (Table 1). DREAMM-2 was an open-label, two-arm, phase 2 study enrolling adult patients with RRMM who had already received three or more lines of therapy. The most common grade 3–4 adverse events in the safety population were thrombocytopenia and keratopathy. Finally, DREAMM-6 is an ongoing phase I/II, two-part, two-arm study evaluating the safety, tolerability, and clinical activity of belantamab mafodotin in combination with bortezomib/dexamethasone (BorDex-Vd) and lenalidomide/dexamethasone in previously treated patients with more than one line of therapy (Table 1). In particular, the data refer to the combination of belantamab mafodotin with BorDex. Part 1 (dose escalation) and Part 2 (dose expansion) evaluated belantamab mafodotin (2.5 and 3.4 mg/kg) given as a SINGLE (day 1) or SPLIT (split equally on days 1 and 8) in combination with BorDex. The multimodal mechanism of action, efficacy, and safety profile of belamaf, as well as preclinical data, suggest a possible synergy with standard-of-care agents and a potential benefit in combination with IMiDs and PIs. The primary endpoints are safety, tolerability, and efficacy. The most frequently reported adverse events are corneal events (including keratopathy, blurred vision, and dry eye) and thrombocytopenia, which are clinically manageable. Preliminary data show that the combination of belantamab mafodotin and BorDex has an acceptable safety profile [56].

The reduction of BCMA expression or mutation in the BCMA gene placed in the 16p locus is a possible mechanism of tumor escape. In MM subjects undergoing a reduction of functional BCMA, a prolonged treatment may not be possible. It is not known if BCMA expression would return to a normal value after a certain interval after anti-BCMA treatment [74].

## 7. Future Perspectives

**Figure 3:**
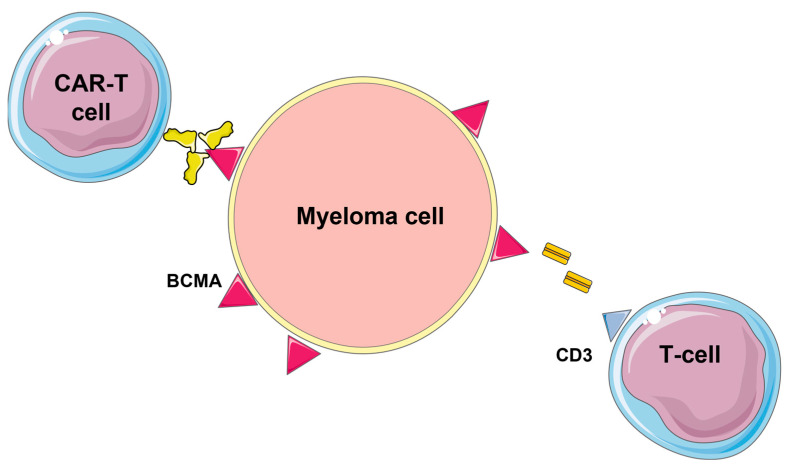
Possible immunotherapeutic options to treat MM.

The list of possible future immunotherapeutic agents with promising results has increased significantly in recent years (Figure 3); however, this disease remains incurable. A solution could be the use of combination therapies that have shown good results in terms of efficacy and survival [1]. A new potential target could be BCMA; several therapies have been approved for this receptor and others are in clinical development. These include CAR-T cell therapies (idecabtagene vicleucel and ciltacabtagene autoleucel) or BsAbs CC-93269, REGN5458, teclistamab, and elranatamab. In a first human phase I study by D’Souza A et al. in patients with RRMM, BCMA-targeted therapy with T-BSAB ABBV-383 was shown to be well tolerated at all doses administered. The ABBV-383 dosing schedule was once every 3 weeks with good patient adherence. Finally, ABBV-383 has standard availability, unlike CAR-T cell therapies, and a shorter hospitalization time than other BCMA and CAR-T cell therapies. The limitations of this study are related to the small number of patients enrolled [75]. Another ongoing phase 1/2 study (NCT03761108) involves REGN5458, which is a bispecific BCMAxCD3 antibody. This was studied for progressive RRMM, for double or triple patients refractory or intolerant to previous lines of systemic therapy, including an inhibitor of the proteasome, an immunomodulatory agent, and an anti-CD38 antibody. In this study, REGN5458 showed profound and lasting responses with a manageable safety profile [76]. In vitro, the BCMAxCD3 bsAb effectively caused polyclonal T-cell destruction of primary plasma cells and MM cell lines presenting a range of BCMA cell surface densities. In vivo, the BCMAxCD3 bsAb reduced the proliferation of human MM tumors in animal experimental models and displayed powerful combinatorial effectiveness with programmed cell death protein 1 blockade. BCMAxCD3 administration to monkeys was tolerated, causing a reduction in BCMA+ cells and slight inflammatory reactions. The anti-MM efficacy of the BCMAxCD3 bsAb was evaluated with BCMA-specific CAR T cells having a BCMA-binding single-chain variable fragment originating from REGN5458. Both BCMAxCD3 and anti-BCMA CAR T cells displayed comparable targeted cytotoxicity of MM cell lines and primary MM cells. Furthermore, BCMAxCD3 quickly cleared established systemic MM tumors, whereas CAR T cells cleared tumors with lengthier kinetics. These findings indicate that the BCMAxCD3 bsAb exerts its therapeutic actions by engaging T cells already in place at the tumor site, whereas anti-BCMA CAR T cells need time to transfer to the tumor site and proliferate before initiating anti-myeloma effects [77].

As for clinical usage, 167 patients with RRMM across phase 1 and 2 trials have received at least one dose of REGN5458. ORR was higher for phase 1 patients treated at ≥200 mg dose levels than for patients treated at doses <200 mg (75% versus 40.8%). Thus, REGN5458 shows promising efficacy in patients with heavily pretreated RRMM [78].

In another study by Moreau et al., teclistamab was evaluated [79,80]. It is a bispecific T-cell redirect antibody that targets both CD3 expressed on the surface of T cells and BCMA on the surface of myeloma cells, thereby mediating activation of T cells and subsequent lysis of BCMA-expressing myeloma cells. In the dose-defining phase 1 study [79,80], teclistamab showed promising efficacy in patients with RRMM. The recommended dose is a weekly subcutaneous injection of 1.5 mg/kg. The mechanism of action of teclistamab is based on a double binding site that targets both CD3 expressed on the surface of T cells and BCMA+ myeloma cells [81]. This effect occurs regardless of the specificity of the T-cell receptor or class I major histocompatibility complex molecules on the surface of myeloma cells [81]. At present, no CD3 redirection therapy has been approved for the treatment of myeloma. Although it is difficult to compare therapies with different mechanisms of action, the response rates observed with teclistamab (63%) can be compared with belantamab mafodotin, which has an overall response rate of 31% in patients with triple class refractory disease [55]. The overall response rates of 67 to 83% have been observed with approved CAR-T cell therapies in patients undergoing apheresis. However, CAR-T cell therapy requires patients to have access to specialized care centers and have to wait a minimum of 4 weeks for production, whereas teclistamab is readily available and has been associated with a rapid onset of response after about 1 month of treatment [82,83]. Several bispecific T-cell activators (TCEs) are in clinical development for multiple myeloma (MM). In the study by Abrams RE et al., both computational and experimental programs were used to investigate how a novel trispecific TCE enhances T cell activation, proliferation, and cytolytic activity against MM cells. In addition to binding CD3 on T cells and CD38 on tumor cells, the trispecific TCE binds CD28, which acts both as a co-stimulator for T cell activation and as an additional tumor target. The trispecific antibody binds to the CD28 receptor on CD28-expressing T cells and MM cells and then provides co-stimulation to all T cells, potentially activating a larger pool of T cells to kill the tumor [84,85,86]. By comparing simulations of the trispecific TCE with a comparable bispecific molecule without CD28 targeting, we find that CD28 offers a significant advantage at lower doses. This finding may be more relevant to the clinical active dose than predictions for higher doses [87].

## 8. Conclusions

The introduction of mAbs to the treatment of MM has already changed clinical practice in patients with RRMM, leading to better outcomes [2]. Additionally, BCMA represents a new promising target for myeloma therapy. Three therapies (belantamab mafodotin and two CAR-T cell therapies, idecabtagene vicleucel and ciltacabtagene autoleucel) targeting the BCMA have been approved for the treatment of MM patients who have previously received immunomodulatory agents, proteasome inhibitors, and anti-CD38 antibodies. Bispecific antibodies targeting tumor antigens and activation of T-cell receptor signaling have shown some clinical efficacy, and trispecific antibodies represent a promising platform for cancer immunotherapy. The list of possible future immunotherapeutic agents that are showing promising results has increased significantly in recent years, thus giving new hope to people suffering with MM.

## Figures and Tables

**Table 1:** Monoclonal antibody types and related studies for treatment of RRMM.

Mab Class	Molecule (Targets)	Phase	Study	Treatment	Toxicities (>_G3)
Naked	Isatuximab (anti-CD38)	I	TCD11863 [1] NCT01749969, open label, dose escalation study	Isa (5 or 10 mg/kg [Q2W] or 10 or 20 mg/kg [QW] for 4 weeks) + R 25 mg (days 1–21) and d 40 mg (QW)	Neutropenia (60%)
Naked	Isatuximab (anti-CD38)	I	TCD14079 [2] NCT02283775	Isa monotherapy ev QW or Q2W	Neutropenia (84%)
Naked	Isatuximab (anti-CD38)	II	NCT01084252 [1], safety and progression-free survival	Isa-d	
Naked	Isatuximab (anti-CD38)	II	NCT02514668 [1], safety and progression-free survival	Isa	
Naked	Isatuximab (anti-CD38)	III	ICARIA-MM [53] NCT029990338, randomized, multi-center open-label study	Isa-Pd (10 mg/kg + p 4 mg + d 40 mg) or Pd (p 4 mg + d 40 mg)	Neutropenia (85%)
Naked	Isatuximab (anti-CD38)	III	IKEMA [54] NCT02514668 prospective, randomized, open-label study	Isa-Kd vs. Kd	Respiratory infections (32.2%)
ADC	Belantamab mafodotin (anti-BCMA, monomethyl auristatin F payload)	I	DREAMM-1 [1] NCT02064387	Belamaf single agent	Thrombocytopenia (35%); keratopathy (14%)
ADC	Belantamab mafodotin (anti-BCMA, monomethyl auristatin F payload)	II	DREAMM-2 [55] NCT03525678	Belamaf single agent	Thrombocytopenia (20%); keratopathy (27%)
ADC	Belantamab mafodotin (anti-BCMA, monomethyl auristatin F payload)	I/II	DREAMM-6 [56] NCT03544281	Belamaf-Vd	Thrombocytopenia (61%); keratopathy (56%)

Isa = isatuximab, R = lenalidomide, d = dexamethasone, K = carfilzomib, P = pomalidomide, V = bortezomib.

## Data Availability

Not applicable.
